# SICEM: A Generation Approach of Band Combination for Hyperspectral Imagery Reconstitution Based on Space and Information Analyses

**DOI:** 10.1155/2021/8178495

**Published:** 2021-09-18

**Authors:** Nian Chen, Kezhong Lu, Hao Zhou

**Affiliations:** ^1^School of Big Data and Artificial Intelligence, Chizhou University, Chizhou 247000, China; ^2^College of Computer Science and Technology, Nanjing University of Aeronautics and Astronautics, Nanjing 211106, China; ^3^College of Computer and Information Science, Southwest University, Chongqing 400715, China

## Abstract

A band selection algorithm named space and information comprehensive evaluation model (SICEM) is proposed in this paper, which reconstitutes the hyperspectral imagery by building an optimal subset to replace the original spectrum. SICEM reduces the dimensions while keeping the vital information of an image, and these are accomplished through two phases. Specifically, the improved fast density peaks clustering (I-FDPC) algorithm is employed to pick out the scattered bands in geometric space to generate a candidate set *U*at first. Then, we conduct pruning in *U*through iterative information analysis until the target set Ωis built. In this phase, we need to calculate comprehensive information score (CIS) for every member in *U*after assigning weights to the amount of information (AoI) and correlation. In each iteration, the band with highest score is selected into Ω, and the ones highly related to it will be removed out of *U*via a threshold. Compared with the four state-of-the-art unsupervised algorithms on real-world HSI datasets (IndianP and PaviaU), we find that SICEM has strong ability to form an optimal reduced-dimension combination with low correlation and rich information and it performs well in discrete band distribution, accuracy, consistency, and stability.

## 1. Introduction

Hyperspectral imagery (HSI) is a combination of spectral detection technology and computer generated imagery (CGI), and by analyzing the data collected by sensors, it can help us grasp the characteristics of objects, as well as the change regularity of spectrum without direct contact. Since any pixel can be described from the perspective of space and spectrum, we usually represent HSI as a three-dimensional cube, that is, on the spectral dimension, each band corresponding to a 2D image. Due to the different degree of absorption and reflection of an object surface against electromagnetic waves with various wavelengths, spectra are distributed on hundreds or even more intervals continuously, and the bandwidth is gradually narrowing along with the development of acquisition instrument (generally less than 10 nm, even 1–3 nm). Nowadays, HSIs have been widely applied for data analyses in many application fields, such as mineral exploration [1], environmental and atmospheric monitoring [2, 3], and agricultural information services [4]. Compared with color image and multispectral image, more information can be recorded in HSI owing to its high resolution, which is conducive to making target recognition more precisely. However, too many similar or overlapping bands also make data complexity increased, so high dimensionality and information redundancy have become the obstacles to calculation and storage. High correlations existed among some adjacent bands probably causing “Hughes phenomenon” [5], and the recognition accuracy increases first and then decreases along with data complexity. Therefore, it is necessary to preprocess the spectrum, including noise removal and redundancy reduction, which can effectively cut down the operation costs and improve the processing speed.

There are two ways to achieve dimensionality reduction for HSI, i.e., band extraction and band selection (BS) [6–9], and according to the data structure and distribution, we may adopt linear or nonlinear approach to realize reduction. Band extraction projects the all-bands into a low-dimensional subspace to form a simplified representation to take place of original features; however, it will lead inherent features of information to change. Some typical technologies include singular spectrum analysis [10] and sparse representation [11]. In contrast, the task of BS is to find out a band combination with rich information, low correlation, and good discrimination, and usually we achieve this via a certain BS algorithm. Moreover, evaluation criterion measures the substitution effect of subset by using embedded, filtered, or encapsulated methods.

Space and information comprehensive evaluation model (SICEM) is extremely suitable for BS. The geometric properties can be effectively utilized for preliminary filtering, whereas information analysis makes more rich-information bands retained to achieve further selection. Recently, the author team has designed a TLS strategy for BS [12] and achieved good results.

Evidently, a single-phase selection approach cannot give consideration to representativeness and high discrimination at the same time; in other words, it is unlikely for clustering-based BS algorithm, such as fast density peaks clustering (FDPC), to pick out the central bands and boundary ones in a spectral interval simultaneously. Therefore, some rich-information bands are probably missed owing to the lack of evaluation to amount of information (AoI). In view of defect of FDPC, the proposed algorithm employs some measurements to improve its performance. The main contributions that have been made are as follows:

1. We make a coarse-grained BS from the perspective of spatial position first and then carry out pruning to get final outputs according to AoI and information correlation. For the selected bands, the former can make them distributed as discretely as possible in the spectrum to decrease spatial redundancy, while the latter ensures that rich and highly independent information is contained in them.
2. Improved FDPC (I-FDPC) overcomes the limitation that the original algorithm tends to select in high-density areas excessively and chooses high-quality elements including cluster centers and boundary nodes in clusters with different sparsity. In addition to scale normalization, we adopt parameter (cutoff distance) self-adaption to make BS more efficient.
3. Calculating comprehensive information score (CIS) after weights assigned to Shannon entropy (SE) and average Jensen-Shannon divergence (AJSD), both AoI and correlation between pairwise bands are treated as a whole, and it helps us evaluate a band from the view of information.

The remaining sections are organized as follows. In Section 2, we will introduce some related research progress about BS technologies in recent years. In the following section, principles of FDPC, I-FDPC, SE, and AJSD analyses will be presented in detail, respectively. In Section 4, we utilize SICEM to realize BS based on the measurement of spatial position and spectral information and give detailed algorithm flow. Based on real-world HSIs, a series of experiments and comparative analyses are conducted to prove the efficiency of the proposed algorithm, and we arrange these in Section 5. At last, some relevant conclusions are given.

## 2. Related Work

As mentioned previously, it is an effective way to achieve dimensionality reduction for HSI via BS, and it can not only cut down the storage and computing consumption for subsequent operation, but also retain the vital properties of spectrum owing to no mathematical transformation. According to the usage of labeled band, BS algorithms are categorized into supervised, semisupervised, and unsupervised methods. If we have mastered the facts that various materials reflect and absorb the electromagnetic waves, establishing a spectral dictionary can provide us more experiences to select the band effectively. However, due to too many kinds and quantities of materials, as well as the huge workload of tagging, this work is difficult to complete. Unsupervised method [13, 14] analyzes the distribution characteristics of bands and the relationships among them and has more adaptability and flexibility to various application scenarios.

### 2.1. Overview of BS Algorithm

Unsupervised BS does not require any labeled sample but seeks an optimal subset to replace the whole spectrum. For example, the ranking-based method prioritizes bands in accordance to a certain criterion and selects top-ranked ones, classical algorithms as constrained band selection (CBS) [15] and maximum variance principal component analysis (MVPCA) [16], etc. Clustering-based method groups the samples by similarity measurement firstly and then picks out the valuable ones in each cluster. Clustering can be implemented through a variety of ways, and some typical approaches (corresponding algorithms), such as the hierarchical-based WaLuDi [17], CURE [18], and partition-based k-Means [19], FCM [20], density-based AP [21], DBSCAN [22], FDPC [23] have been successfully applied to BS. Moreover, some algorithmic ideas [24–26] proved to be effective in other fields and can also be migrated to this application.

Undoubtedly, BS will bring extra computation costs, and there are some errors between its outputs and the all-bands. Nevertheless, these do not reduce the necessity of BS, for it plays an important role in eliminating redundancy and improving the speed of subsequent image procession. Evidently, it is not recommended to get an optimal one by comparing all band combinations due to a lot of computation produced.

Generally, ranking-based method can find out the bands with large AoI, while high correlations are inevitable owing to differences among the bands neglected. Clustering-based method has strong ability to establish a discriminative and representative combination, because the similarity of maximum intracluster and minimum intercluster guarantees low redundancy between pairwise bands selected from different clusters. In recent years, some intelligent search algorithms have been applied to BS in order to improve its parallelism, robustness, and universality, such as genetic algorithm [27], particle swarm optimization [28], and artificial bee colony algorithm [29].

At present, the research hot-spots of BS mainly focus on the following aspects. (1) How to improve the search speed and validity of algorithm output. Generally, selection through two phases can get more concise and valuable bands, although it will lead additional computation concurrently. (2) Enhancing the generalization ability of an algorithm in various data environments is another important issue, so it is a core work to replace manual setting with parameter adaptation. (3) It is the trend of hyperspectral dimensionality reduction that mixes BS algorithm and classifier model together.

### 2.2. Research Progress of FDPC

Observed from the geometric distribution, high-density areas are separated by low-density ones. As a synthesis algorithm that employs ideas of ranking-based and density-based, FDPC obtains the globally optimal solution through a few parameters, simple process (no iteration required, and no initialization to cluster centers). Especially, FDPC has the ability to find arbitrary shaped cluster rather than just spherical region, which makes it more adaptable to various data distributions. However, when there are no density peaks or fake peaks, the algorithm cannot play its advantage. In addition to its application in HSI, we also have successfully adopted FDPC to solve other classification problems, such as natural language processing [30] and biodata analysis [31, 32]. Nevertheless, there are still some technical obstacles to be solved, including time/space complexity reduction, adaptive ability of the parameter enhanced, and accuracy and robustness improved. In the rest of this section, we present some optimization practices.

The time complexity of FDPC is *O*(*n*^2^), where *n* is the number of samples, and the algorithm is ill-suited to deal with large-scale data accordingly. In order to achieve lower complexity, we use parallel processing or grid treatment to cut down the execution time. For example, EDDPC [33] selects the seeds needed for Voronoi segmentation and groups the data, and then two MapReduce tasks are employed to calculate the densities and distances in each group in parallel. LSH-DDP [34] uses local sensitive hash to partition the data and performs local computation within a partition, and the final output is obtained through aggregation. In comparison, the speed of LSH-DDP has doubled that of EDDPC. DGB [35] and DPCG [36] utilize grid technology to divide the sample space into multiple cells according to dimensions and use the distance between cells to replace Euclidean distance so as to speed up the implementation of algorithms.

As we know, the cutoff distance is the only parameter for the algorithm adjusted that determines the statistical result of local density and affects the constitution of similarity matrix directly. Parameter self-adaption can reduce the probability of error occurrence and make the algorithm more universal. For example, ADPC-KNN [37] designs a density calculation method based on KNN and Gaussian kernel function. DHeat [38] overcomes the limitation brought by fixed cutoff distance, so it processes the data set with high-dimensions and fake peaks better.

## 3. Approaches

### 3.1. BS Based on Spatial Dispersion

#### 3.1.1. Algorithm Theory of FDPC

The premise of using FDPC is that the data distribution meets the following two assumptions. In each cluster, firstly, the center has the maximum local density, and secondly, the distance between the center and the node with higher density is relatively large. FDPC employs two variables to describe the spatial characteristics of a node, i.e., local density *ρ* and relative distance *δ*, and both of them are constrained by cutoff distance *d*_*c*_.

We represent a hyperspectral image I in spectral and pixel space, respectively,*I*=(*b*_1_, *b*_2_,…, *b*_*L*_)=(*x*_1_, *x*_2_,…, *x*_*N*_), where the numbers of bands and pixels are denoted as *L* and *N*. *b*_*l*_={*b*_*l*_^*i*^*|i*=1,2,…, *N*} is the responses of all pixels to *l*_th_ band, which also can be regarded as a projection of HSI on *b*_*l*_, and *x*_*t*_={*x*_*t*_^*i*^*|i*=1,2,…, *L*} is a reflection of *t*_th_ pixel against different bands. Generally, we build an initial similarity matrix *S*=*R*^*L*×*L*^ and define *d*_*ij*_ as the distance between two bands based on matrix *S*, as shown in the following equation:

(1) $$\begin{array}{c} d_{ij}=\frac{\sqrt{S_{ij}}}{L},\\ S_{ij}={\left\|R_{i}-R_{j}\right\|}^{2}=\sum_{n=1}^{N}{\left(R_{ni}-R_{nj}\right)}^{2}. \end{array}$$

We commonly use Euclidian distance between vectors *R*_*i*_ and *R*_*j*_ to describe the similarity of pairwise band in practice. Consistent with our understanding, a closer interband distance corresponds to a higher possibility of redundancy, because studies have demonstrated that the reflection and absorption of electromagnetic waves with adjacent frequencies are highly overlapped.

The local density *ρ*_*i*_ is expressed as

(2) $$\begin{array}{c} \rho_{i}=\sum_{j=1,j\neq i}^{L}\chi \left(\text{dist}\left(b_{i},b_{j}\right)-d_{c}\right),\\ \chi \left(x\right)=\left\{\begin{array}{cc} 1, & x\leq 0,\\ 0, & x>0. \end{array}\right. \end{array}$$

For *b*_*i*_, FDPC counts the nodes in its neighborhood to get *ρ*_*i*_. Obviously, the indicator function *χ*ignores the influence of node position on density, and *ρ*_*i*_ increases by one as long as *d*_*ij*_ < *d*_*c*_. As an effective solution, the use of Gaussian kernel function *R*_*G*_(*x*, *y*)=exp(−(‖*x* − *y*‖^2^/2*σ*^2^)) makes *ρ*_*i*_ depend on not only the size of *d*_*c*_ but also the compactness of nodes.

(3) $$\begin{array}{c} \rho_{i}=\sum_{j}e^{-{\left(d_{ij}/d_{c}\right)}^{2}}. \end{array}$$

The experience shows that FDPC performs well when *d*_*c*_ is set to 1%–2% of interband distances sorted in descending order. Inappropriate *d*_*c*_may cause meaningless statistics or produce false outliers, so it is necessary to initialize *d*_*c*_ as precisely as possible through some reasonable approaches. For example, ADPC-KNN calculates the density of a node by using KNN, as shown in the following equation:

(4) $$\begin{array}{c} \rho_{i}=\sum_{j\in {\text{KNN}}_{i}}e^{-{\left(d_{ij}/d_{c}\right)}^{2}},\\ d_{c}=\frac{1}{N}\sum_{i=1}^{N}\theta_{i}^{k}+\sqrt{\frac{1}{N-1}\sum_{i=1}^{N}{\left(\theta_{i}^{N}-\frac{1}{N}\sum_{i=1}^{N}\theta_{i}^{k}\right)}^{2}}, \end{array}$$

where *θ*_*i*_^*k*^=max_*j*∈kNN_*i*__(*d*_*ij*_).

Next, the definition of *δ*_*i*_ is given as follows:

(5) $$\begin{array}{c} \delta_{i}=\left\{\begin{array}{cc} \underset{j:\rho_{i}<\rho_{j}}{\min}\left(\text{dist}\left(x_{i},x_{j}\right)\right), & \exists j\;s.t.\;\rho_{i}<\rho_{j},\\ \underset{j}{\max}\left(\text{dist}\left(x_{i},x_{j}\right)\right), & \text{otherwise.} \end{array}\right. \end{array}$$

**Figure 1:**
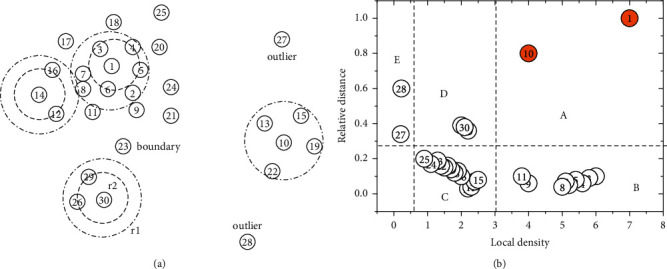
Nodes distribution and decision graph. (a) Spatial distribution of nodes. (b) Decision graph corresponding to (a).

Let *δ*_*i*_ be the distance that between *b*_*i*_ and the node farthest from it, only when *b*_*i*_ has the maximum local density. Generally, if *b*_*i*_ is not corresponding to peak density, we get *δ*_*i*_ by calculating the distance between it and the nearest node among all of higher density ones. In Figure 1(a), 30 nodes are unevenly distributed on a plane, and a decision graph is established for intuitive analysis by taking *ρ*and *δ*as axes. As shown in Figure 1(b), the cluster centers are usual outliers, and we consider the nodes in regions *A*, *D*, and *E* as centers of dense cluster, sparse cluster, and single band cluster, respectively.

However, nodes close to the horizontal axis are unlikely to be independent centers due to low discrimination caused by excessive concentration. According to *δ*, most of them in region *B* are nonboundary nodes and those in region *C* are boundary ones. Initializing the number of clusters beforehand, *FDPC* takes the density-peak node in each cluster as condensation point, and the rest are allocated to the nearest and higher density areas. In addition, it is illustrated that the algorithm has strong noise resistance capability through decision graph, that is, finding out the interference easily. Obviously, the isolated nodes near vertical axis, such as nodes 27 and 28, are probably noises.

For *b*_*i*_ ∈ *I*, FDPC uses the inner product *γ*_*i*_=*ρ*_*i*_ × *δ*_*i*_ to reflect its spatial characteristics and prioritize *γ* in descending order for getting a sequence *γ*_1_ > *γ*_2_ > ···>*γ*_*m*_ > *γ*_*m*+1_ > ···>*γ*_*L*_. On this basis, we form a candidate set by grouping *U*={*b*_spt(*γ*_1_)_, *b*_spt(*γ*_2_)_,…, *b*_spt(*γ*_*m*_)_}, where spt(*γ*_*i*_) is the subscript of band corresponding to *γ*_*i*_ and *m* is the number of required bands. To ensure the representativeness of FDPC outputs, only exemplar in each cluster will be picked out. Therefore, the algorithm prefers the nodes in high-density region rather than class boundary, which probably leads to the loss of vital information.

#### 3.1.2. Improved FDPC

Due to technical limitations of FDPC, the effects of its outputs often lag behind our expectation. Hence, I-FDPC carries out two improvements on the basis of original algorithm. *ρ* has a greater impact on prioritization compared with *δ*, so the nodes with high-densities are easily placed at the front of *γ*sequence, which makes them more attractive to FDPC. For example, supposing that we have to prioritize four nodes in Figure 1(b), i.e., node 8 (*ρ*_8_=5, *δ*_8_=0.08, *γ*_8_=0.4), node 15 (*ρ*_15_=2.5, *δ*_15_=0.1, *γ*_15_=0.25), and node 23 (*ρ*_23_=1.2, *δ*_23_=0.2, *γ*_23_=0.24), node 8 has the highest priority (*γ*_8_ > *γ*_15_ > *γ*_23_), which is caused by its highest *ρ*in spite of *δ*_8_ < *δ*_15_ < *δ*_23_. However, it does not mean that *ρ*always plays a decisive role to priorities. Although the local density of node 10 (*ρ*_10_=4, *δ*_10_=0.8, *γ*_10_=3.2) is smaller than that of node 8, the outstanding advantage of *δ* also makes it the preferred one. Commonly, both *ρ* and *δ* are normalized to interval (0,1] to realize consistent metric.

(6) $$\begin{array}{c} \tilde{\rho_{i}}=\frac{\rho_{i}-\rho_{\min}}{\rho_{\max}-\rho_{\min}},\\ \tilde{\delta_{i}}=\delta \frac{\delta_{i}-\delta_{\min}}{\delta_{\max}-\delta_{\min}}. \end{array}$$

Normalization can weaken but not eliminate the dominant role of density. (*γ*_10_(0.456) > *γ*_8_(0.057) > *γ*_15_(0.035) > *γ*_23_(0.034)) is the node priorities after transformation, and obviously, normalization does not change the previous results. Hence, we should further adopt parameter self-adaptation to improve the performance.

For the sake of simplicity, the empirical way usually sets *d*_*c*_with fixed size, but it is inefficient when processing dataset with special forms, especially uneven density distribution. Undoubtedly, it is unfair to the nodes that are located in low-density clusters or boundary if great-sized *d*_*c*_ is adopted. As illustrated in Figure 1(a), we calculate the densities of nodes at three representative positions, i.e., node 1 (center of dense cluster), node 14 (boundary), and node 30 (center of sparse cluster). When *d*_*c*_=*r*_1_, we get *ρ*_1_=7, *ρ*_14_=3, *ρ*_30_=2; however, if *d*_*c*_ is turned to *r*_2_(*r*_2_ < *r*_1_), *ρ*_1_=4, *ρ*_14_=2, *ρ*_30_=2. Obviously, with the decrease of *d*_*c*_, the density advantage of node 1 is greatly weakened, while node 14 and node 30 are slightly or not affected, respectively. This shows that a proper initialization of *d*_*c*_ can effectively control the outputs of FDPC. Hence, to make more nodes generated from the sparse regions rather than dense ones, we initialize *d*_*c*_ according to *m*.

(7) $$\begin{array}{c} d_{c}=d_{c-0}\times \left(-\;\log_{2}\frac{m}{L}\right). \end{array}$$

In equation (7), *d*_*c*−0_ is the baseline value of cutoff distance. With the increase of *m*, *d*_*c*_ is going smaller and *ρ* decreases synchronously. Especially, if each node corresponds to a cluster, i.e., *m*=*L*, we get *d*_*c*_=0, *ρ*=0. In this case, density statistic is meaningless, and I-FDPC will eventually fail.

### 3.2. Band Information Evaluation

Besides spatial position, AoI is another important metric to BS, and it is generally believed that the greater the uncertainty of band status is, the more information it contains. In this paper, we employ SE to measure AoI contained in a band and evaluate the independence of information within spectrum via AJSD.

An event with large entropy corresponds to a strong uncertainty, and it also means that more information can be provided for judgement. Assuming that the band *b*_*i*_gets different values with various probabilities, its SE is defined as equation (8), where *b*_*ik*_ is the *k*_th_ possible value of *b*_*i*_.

(8) $$\begin{array}{c} H\left(b_{i}\right)=-\sum_{k}p\left(b_{ik}\right)lb\left(p\left(b_{ik}\right)\right). \end{array}$$

SE describes AoI within a band, but it cannot reflect the correlations between information. KL divergence (KLD) makes up for the lack of SE, so by employing it, we remove some bands with redundant information and prevent high-related bands from being selected excessively.

Denote two discrete probability distributions of random variable *X* as *P*(*x*) and *Q*(*x*), and accordingly, KLD of *P* to *Q* is

(9) $$\begin{array}{c} D_{\text{KL}}\left(\left.P\right\|Q\right)=\sum_{x\in X}P\left(x\right)\times \;\ln\frac{P\left(x\right)}{Q\left(x\right)}\text{,} \end{array}$$

where *D*_KL_(*P*‖*Q*) represents the loss caused by fitting the real distribution with theoretical distribution *Q*, and it is nonnegative and does not satisfy some properties of distance. Evidently, the higher the similarity is, the smaller the KLD value got. When these two distributions are exactly the same, we get *D*_KL_(*P*‖*Q*)=0. Due to the asymmetry of KLD, that is, *D*_KL_(*P*‖*Q*) ≠ *D*_KL_(*Q*‖*P*), JSD is adopted to solve the problem pertinently.

(10) $$\begin{array}{c} D_{\text{JS}}\left(\left.P\right\|Q\right)=\frac{1}{2}\left(D_{\text{KL}}\left(\left.P\right\|\frac{P+Q}{2}\right)+D_{\text{KL}}\left(\left.Q\right\|\frac{P+Q}{2}\right)\right). \end{array}$$

After obtaining the information correlation between any pairwise bands in spectrum, a *m* × *m* JSD matrix *M*_*JS*_is established as

(11) $$\begin{array}{c} M_{\text{KL}}=\left[\begin{array}{cccc} 0 & \cdot \cdot \cdot & \cdot \cdot \cdot & D_{\text{JS}}\left(\left.b_{1}\right\|b_{m}\right)\\ D_{\text{JS}}\left(\left.b_{2}\right\|b_{1}\right) & 0 & \; & \cdot \cdot \cdot \\ \cdot \cdot \cdot & \; & 0 & D_{\text{JS}}\left(\left.b_{m-1}\right\|b_{m}\right)\\ D_{\text{JS}}\left(\left.b_{m}\right\|b_{1}\right) & \cdot \cdot \cdot & \cdot \cdot \cdot & 0 \end{array}\right]. \end{array}$$

For any *b*_*i*_, we use AJSD, i.e., $\bar{D_{\text{JS}}\left(b_{i}\|;\right)}=1/\left(m-1\right)\sum_{k=1,k\neq i}^{m}D_{\text{JS}}\left(b_{i}\|b_{k}\right)\in \left[0,1\right]$ to express the average loss of fitting it. A small AJSD implies that the information contained in *b*_*i*_ is highly redundant with other bands. On the contrary, larger *b*_*i*_ is synonymous with strong information independence and is not easily replaced.

## 4. Optimal Combination Based on SICEM

### 4.1. Weighted Spectral Information Measurement

I-FDPC achieves preliminary dimensionality reduction from the perspective of geometric screening. However, it is one-sided to measure a band without considering information, so we introduce CIS that performs weighted summation of AoI and AJSD. According to CIS, we conduct a further pruning to the outputs of I-FDPC, and the informative and low information-redundancy combination is generated to take place of original spectrum.

Clearly, any band suited to the optimal combination should have not only large AoI, but also low spectral similarity. We use coefficient-weighted to allocate the influence degree of these two factors, and the specific weights ought to be determined according to actual band distribution.

(12) $$\begin{array}{c} \text{CIS}\left(b_{i}\right)=\omega_{1}\cdot H\left(b_{i}\right)+\omega_{2}\cdot \bar{D_{\text{JS}}\left(\left.b_{i}\right\|;\right)},\\ \omega_{1}+\omega_{2}=1. \end{array}$$

### 4.2. Design Idea and Implementation Flow

**Figure 2:**
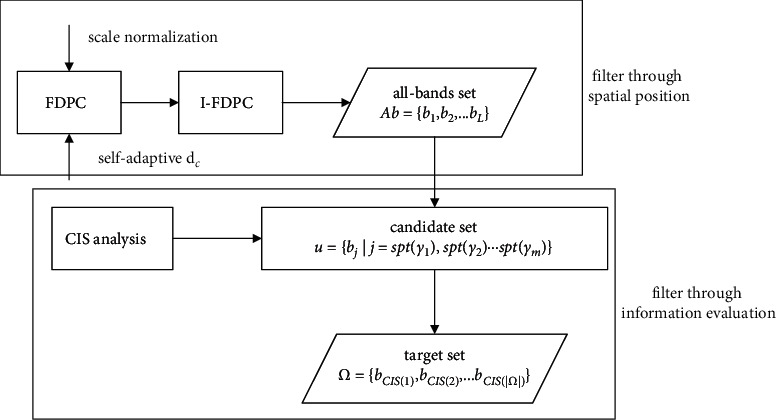
Diagram of SICEM idea.

The diagram of SICEM idea is shown in Figure 2, and there are two highlights existing in the algorithm design. One is double filtering, which aims to build a simplified representation for sample in low-dimension space. To achieve this, we carry out BS from the view of spatial position and information evaluation in turn. The other is to integrate AoI and information correlation by using CIS, which makes the information measurement to band more comprehensive.

Specifically, the candidate set *U*={*b*_*j*_*|j*=spt(*γ*_1_), spt(*γ*_2_),…, spt(*γ*_*m*_)} is built followed by target set initialization Ω=∅. To avoid repetition, we will not explain the generation of *U*anymore and just briefly describe the process of building Ω as follows: (1) Sort{CIS(*b*_j_), *b*_j_ ∈ *U*}. (2) Choose *b*_*p*_with highest CIS in the current round to enrich Ω, i.e.Ω=Ω+*b*_*p*_. (3) The remaining bands in *U*are compared with *b*_*p*_one by one, and thus several ones with approximate information are filtered via threshold *ϑ*, *U*=*U* − {*b*_*k*_*|D*_JS_(*b*_*p*_‖*b*_*k*_) < *ϑ*}. (4) Iteratively execute (2), (3) until *U*=∅. Ω={*b*_CIS(1)_, *b*_CIS(2)_,…, *b*_CIS(|Ω|)_} is what we expected, where *b*_CIS(*i*)_ is the band got through *i*-round CIS analysis. We give the pseudocode in Algorithm 1.

In theory, target set is the best if it can achieve the desired accuracy with the smallest |Ω|; we always aim to design an algorithm to find out the optimal Ω. Undoubtedly, the optimal feature combination must correspond to the highest efficiency, so the common expectation of various BS algorithms including SICEM is defined as formula (13), where acc_*F*_is the accuracy based on F-feature representation. Besides accuracy, consistency and stability are also important criteria for algorithm evaluation, and we will discuss them in the following section.

(13) $$\begin{array}{c} \underset{\Omega \subset Ab}{\arg\max}\left(\frac{{\text{acc}}_{\Omega }}{\left|\Omega \right|}\right)=\underset{\Omega \subset Ab}{\arg\min}\left(\frac{{\text{acc}}_{Ab}-{\text{acc}}_{\Omega }}{\left|\Omega \right|}\right). \end{array}$$

### 4.3. Performance Analysis to SICEM

Generating a candidate set produces major costs of SICEM. The time complexity of I-FDPC is *O*(*N* × *L*^2^), which is caused by computing distance of interband to build similarity matrix. In the phase of information evaluation, SICEM needs to obtain CIS of each band in *U*, and it makes consumption of *O*(*m* × *N*). Also, eliminating redundant bands will result in *O*(*m* × *N*). Therefore, the time complexity of SICEM is *O*(*N* × (*L*^2^+*m*)), which is slightly higher than that of I-FDPC, so apparently, the real-time performance of proposed algorithm is not strong to high-resolution images.

As a double filtering approach, the final effect of SICEM depends heavily on outputs of I-FDPC. Thus, the algorithm will be invalid when meeting no peak or fake peak, although it has the ability of getting optimal solutions in global scope. Besides this, we have to initialize parameter *m* in advance instead of relying on automatic aggregation. Moreover, threshold *ϑ* and weight coefficients *ω*_1_, *ω*_2_ still need to be set by experiences, which brings uncertainty to the execution effect of algorithm, although we have limited their range. It is noteworthy that the pruning is not back-traceable; in other words, a band cannot be recovered after being pruned as a redundant one.

In conclusion, SICEM generates a reduced band combination to replace whole spectrum and provides more valuable features for classifier training. The algorithm not only inherits the advantages of I-FDPC, such as no iteration, good at exemplar selection in irregular area, noise insensitivity, self-adaptive cutoff distance, and no initialization to cluster center, but also makes information more critical to further reduction by employing CIS. Compared with other BS algorithms, the prominent advantage of SICEM is that it can describe samples more efficiently with the same number of features, thus making the generalization ability of the classifier stronger.

## 5. Experiments and Discussion

In this section, we conduct a series of experiments on different HSI datasets, and some performance comparisons between SICEM and four unsupervised algorithms using overall accuracy (OA), average accuracy (AA), and Kappa coefficient (KC) are followed. The discussions focus on these topics: (1) spectral distribution of target set formed by different algorithms; (2) influence of some factors, such as the number of selected bands and classification model on HSI recognition performance; (3) stability analysis to SICEM. As preparation, we introduce the relevant contents firstly, including datasets, design of experiments, and indicators for capability comparison, and so on.

### 5.1. Preparation for Experiments

#### 5.1.1. Datasets

Universal real-world HSI datasets derived from remote sensing images, including Indian Pines (IndianP) and Pavia University (PaviaU) (URL: http://www.ehu.eus/ccwintco/index.php?%20title=Hyperspectral_Remote_Sensing_Scenes#Indian_Pines), are employed for experiments, and the essential situations are briefly described in Table 1.

**Figure 3:**
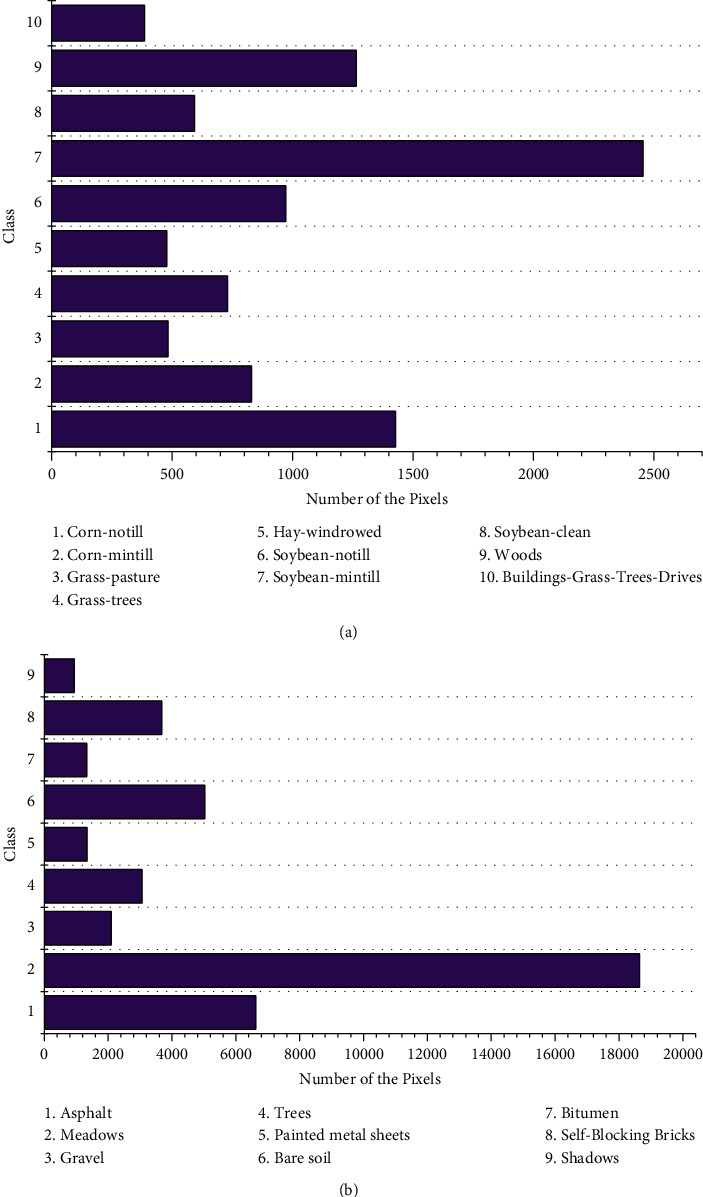
Pixel distribution on different classes of two datasets. (a) IndianP with 10 classes. (b) PaviaU with 9 classes.

Compared with IndianP, clearly, PaviaU has not only higher image resolution, but also fewer bands, and accordingly there are more pixels contained in each land-cover class. Sufficient and evenly distributed samples are helpful in improving the accuracy of recognition, which will be verified in subsequent experiments. Since several miniscale classes in IndianP cannot provide enough samples for classifier training, such as Alfalfa, Grass-pasture-mowed, and Oats, we only retain ten classes in IndianP to make the experimental results more valuable for comparison, as seen in Figure 3(a).

Apart from the difference, there are also some common characteristics with both datasets. First of all, pixels belonging to the same class have similar spectral responses, whereas obvious contrasts exist among distinct classes. Secondly, distribution of pixels among classes is uneven, and it leads to spectral feedbacks mainly concentrated in a few bands. Finally, “different body with same spectrum” or “same body with different spectrum” phenomenon exists in two HSIs, which probably makes errors between the classification results and real values, although some contaminated bands have been removed to ensure the validity of data.

#### 5.1.2. Design of Experiments

To verify the effectiveness of SICEM, MVPCA, WaLuDi, DBSCAN, and I-FDPC are taken as competitors to reconstitute HSIs, respectively. We set the variables and parameters mentioned in Algorithm 1 as follows. *d*_*c*−0_=min*d*_*ij*_, *m* < (*L*/2) (it makes *d*_*c*_ greater than *d*_*c*−0_; in fact, it is better to initialize *m* to about 1/4 of *L*), *ϑ* ∈ (0,0.2]. Furthermore, we assign a higher weight coefficient to AoI, *ω*_1_ ∈ [0.6, 0.8], and set *ω*_2_ ∈ [0.2, 0.4] correspondingly.

We train KNN (*K* = 5) and SVM (RBF kernel function) models with labeled samples. Due to uncertainty of experimental outputs, we average the results of 10 rounds as final to make them more referable and convincing. From IndianP (PaviaU), 30% (10%) samples in every class are picked out randomly, and fivefold cross validation is employed, that is, four-fifths for training and one-fifth for test. When conducting stability test, 10% of the samples in PaviaU will be divided into four subsets, and the stability of SICEM is got by pairwise comparing the features obtained from the above subsets.

#### 5.1.3. Performance Indicators

OA, AA, and KC are commonly used as indexes to evaluate classification effect based on confusion matrix. OA takes an entire test set as the denominator to calculate overall accuracy; however, it cannot reflect the recognition effect of individual class. Different from OA, AA averages the accuracies of multiple classes to represent classification capability. KC is usually employed for consistency check, and in general, a larger KC means that the prediction result is more consistent with ground truth. Specifically, 0.8 > KC > 0.6 means good match, and KC ≥ 0.8 corresponds to perfect match. The sensitivity of an algorithm to data changes is also an important index, and a strong stability indicates that more of the same features can be extracted under dynamic data environment. Supposing that *f*_*i*_, *f*_*j*_ are feature sets obtained on data subset DS_*i*_ and DS_*j*_, we use Jaccard coefficient to measure the similarity of two feature combinations.

(14) $$\begin{array}{c} J\left(f_{i},f_{j}\right)=\frac{\left|f_{i}\cap f_{j}\right|}{\left|f_{i}\cup f_{j}\right|}. \end{array}$$

### 5.2. Results Analysis and Discussion

#### 5.2.1. Distribution of Selected Bands

**Figure 4:**
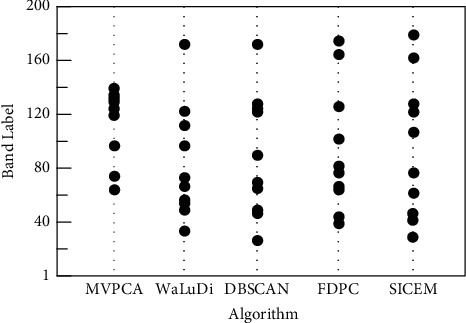
Comparison of BS results using five algorithms on IndianP.

Five algorithms are applied to spectral dimensionality reduction on IndianP, respectively; spatial locations of 10 bands are shown in Figure 4, from which we observe the distribution directly. In theory, if the selected bands are excessively concentrated, the classifier cannot grasp more comprehensive features to promote generalization ability. Therefore, spatial dispersion is an intuitive reflection of band representativeness.

In the interval (120, 140), seven adjacent bands are selected densely by MVPCA, and evidently, these outputs only reflect the importance of bands rather than their representativeness. As mentioned in Section 2, ranking-based algorithm prioritizes the variances to realize BS, so high redundancy is likely to occur owing to the correlations between pairwise bands neglected. However, significant differences do not appear when the rest of algorithms are carried out, and their productions are relatively scattered. Clearly, any clustering-based algorithm also cannot make its outputs uniformly distributed on the entire spectrum; in other words, concentrations presented in some intervals are inevitable. However, this phenomenon is beneficial to machine learning, for the high-density regions of spectrum contain more energy that can help the classifier. Comparatively, the effect of SICEM is slightly better owing to the double filtration employed, and we find that its distribution is wider a little, and the local redundancy is relatively lower.

#### 5.2.2. Accuracy and Consistency

**Figure 5:**
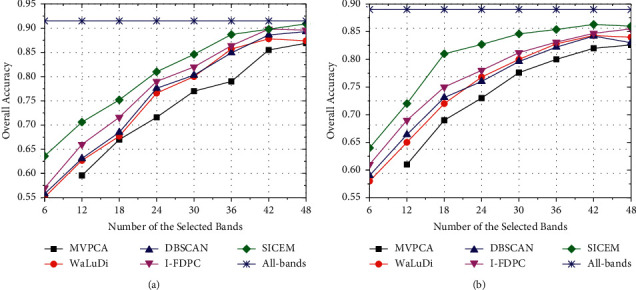
OA curves correspond to different BS algorithms on the IndianP dataset using classifier SVM ((a)) and KNN ((b)).

**Figure 6:**
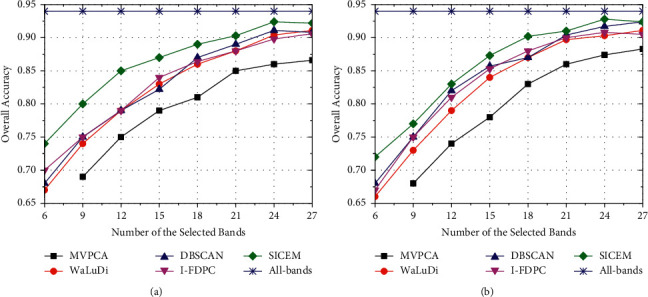
OA curves correspond to different BS algorithms on PaviaU dataset using classifier SVM ((a)) and KNN ((b)).

For each HSI dataset, we set maximum *m* as about 25% of the number of available bands, that is, *m* = 48 for IndianP and *m* = 27 for PaviaU. It is affirmed that the contributions of bands selected by various algorithms to image recognition are unstable, which depends on both classification model adopted and dataset. Even if the model and data environment are exactly the same, the results of each round may not coincide perfectly. Through Figures 5 and 6, we find the following facts:

1. For any algorithm, the increment of OA is synchronized with that of *m*; the improvement, however, changes from fast to slow, even the negative appeared in some cases. Generally, information contained in bands can effectively help the classifier enhance discrimination ability, but redundant selection is not helpful in accuracy promotion. Taking Figure 5(a) as an example, OAs of various algorithms have improved by about 20% with *m* up from 6 to 24 except MVPCA, which proves that the samples are more distinguishable in high-dimensional space. However, OA curves maintain at the current level when we raise *m* from 42 to 48, because similar features have little effect on the evolution of classification model. In addition, excessive selection also increases the computation burden and may cause overfit to make accuracy decline.
2. OA obtained via SVM is superior to that via KNN. Theoretically, SVM seeks a hyperplane that can maximize the margin between two classes, and the class label of a nonclassified sample depends on its position relative to hyperplane. Compared with intracluster samples, support vectors at the boundary are more valuable. Different from the former, KNN uses nearest neighbors voting way to assign label, and *K* affects the ownership of sample ultimately. Generally, the classification errors are mainly caused by fuzzy or noise samples. For HSI application, pixels, especially fuzzy ones, can be well presented if we employ adequate critical bands. SVM makes more effective use of boundary pixels, so it has a better generalization power and stronger noise resistance ability comparatively.
3. The performance of an algorithm is closely related to data environment. Observed from curves, OAs achieved on PaviaU are significantly higher than those achieved on IndianP under the same conditions. For example, the algorithms except MVPCA can achieve 90% or higher accuracy on PaviaU but only about 80% on IndianP, when *m* = 24 (Figure 5(a) and 6(a)). As seen in Figure 3, the class scale of IndianP is much smaller than that of PaviaU. Although we have removed the miniclasses, the classifier still cannot be fully trained owing to insufficient pixels, which makes the performances of various algorithms on IndianP inferior to PaviaU. Hence, excellent BS improves the representation quality of pixels, while enough samples are important support to the required accuracy.
4. The accuracy curve of SICEM is always above that of other competitors. Its advantage is more prominent especially when training with low-dimensional samples, because SICEM can provide more efficient representation of pixels to help classifier promotion. In Figure 6(a), it makes OA close to 75% using 6 bands, which is about 5% higher than that of WaLuDi, DBSCAN, and I-FDPC. However, this superiority is gradually weakened along with more bands added, and there are few differences among their performances when *m* = 27.

Taking 20% of available bands for HSI reconstitution, the corresponding accuracies have achieved a relatively stable level when this proportion is used. AA, OA, and accuracy of single class are given in Tables 2 and 3.

Obviously, the test effects on PaviaU are better, no matter what index above is adopted. Thus, we draw a conclusion that the accuracy depends more on inherent characteristics of HSI, such as complexity of image pattern and noise. In other words, if there are lots of fuzzy-boundary bands or interference waves existing in HSI, the power of algorithm must be weakened. However, those such as I-FDPC, DBSCAN, and SICEM have strong noise resistance, so they may be less affected.

Moreover, some algorithms have ordinary performances on most classes but do well on specific ones, such as MVPCA on Hay-windrowed (Table 2) and DBSCAN on Self-Blocking Bricks (Table 3), because there is a good match degree between the algorithm and data distribution. Similarly, on a few individual classes, accuracies achieved via SICEM also will be less than its competitors. In addition, OAs are better than AAs owing to different calculation way; it is quite evident that a high accuracy on large-scale class will push up OA, such as Meadows (Table 3). Comparatively, AA is not affected by this due to the class scale not involved, and it reflects the recognition ability of classifier on each class.

As last items in above Tables, variance comparison shows that the performance fluctuation of classifier on different classes is smallest if SICEM outputs are employed for pixel representation, which forms a great contrast to effects obtained by using MVPCA.

**Figure 7:**
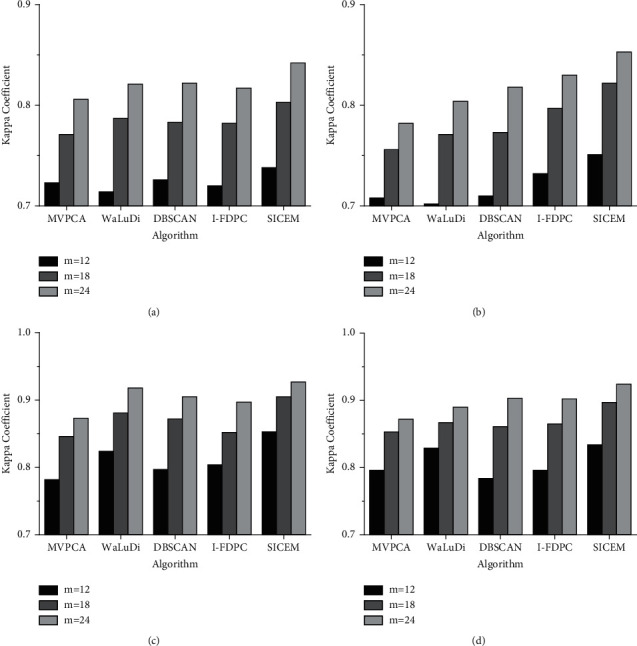
Kappa coefficients obtained through five algorithms under various conditions. (a) (IndianP-SVM), (b) (IndianP-KNN), (c) (PaviaU-SVM), and (d) (PaviaU-KNN) according to the format (dataset-classifier).

As shown in Figure 7, KCs of five algorithms are all greater than 0.7 and even more than 0.9 in some cases. It implies that the classification results are highly consistent with ground truths, and the critical band information contained in image is not lost after dimensionality reduction. Intuitively, KC is directly proportional to the number of selected bands, while the growth rate gradually declines. Just the same principle as the above accuracy analysis, we draw the following conclusions about KC. (1) SVM performs better than KNN with the same conditions, especially on IndianP. (2) We can get higher KCs when taking PaviaU as background. (3) The consistency of SICEM is superior to that of other competitors.

#### 5.2.3. Stability and Iteration

**Figure 8:**
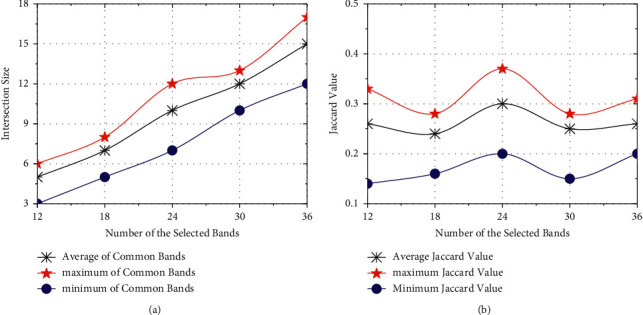
Stability of SICEM varies with the number of bands on PaviaU. (a) Change of intersection size. (b) Using Jaccard index.

It is an effective way to verify the stability of an algorithm by comparing the bands collected on different datasets, and Figure 8 shows the stability test of SICEM on PaviaU. With the increase of selected bands, the intersection size of two band combinations also grows synchronously.  Figure 8(b) illustrates that the average Jaccard index is basically stable around 0.25, which indicates that SICEM has strong ability to cope with the changes of external data environment. Obviously, the probability of selecting exactly same feature based on different sample subsets is small, because any one of redundant bands can achieve the similar effect. Therefore, although Jaccard index is relatively low, it does not mean that the algorithm has poor stability.

As seen in Algorithm 1, similarity threshold *ϑ* determines the iteration rounds of pruning, and we can control the information independence in Ω via it. In practice, since the spectrum has already been screened by I-FDPC, we just need to set *ϑ* smaller to remove a few redundant bands out of *U*. Let |*U*| be 40% of available bands, for this proportion is conducive to the generation of more redundant bands in *U*so as to facilitate the role of pruning. The relationships between threshold intervals and iteration rounds (expressed as maximum and minimum values) are shown in Table 4. Evidently, iteration rounds go down with the increase of *ϑ*, which is completely consistent with the theoretical estimate.

## 6. Conclusions

In this paper, we propose an algorithm named SICEM to build a dimensionality-reduced band set for HSI reconstitution. The algorithm takes the spatial distribution, AoI, and information correlation into account comprehensively and picks out the bands with strong discrimination, low redundancy, and high information through two phases. First, for every member in all-bands set, we employ I-FDPC algorithm to sort their inner products of local density and relative distance in decreasing order, and the top-ranked bands are collected into candidate set. Initialization optimized is done for I-FDPC, and the approaches of normalization and self-adaptive cutoff distance are used, so that the algorithm outputs are scattered rather than concentrated in high-density region. Next, we assign weights to AoI and information correlation and calculate CIS of every band in candidate set. In each round, SICEM retains the current highest-score band and removes those ones, which are highly correlated to it via threshold. Iterate until the candidate set is empty, and the final band combination is formed.

Taking four algorithms as the competitors, we compare SICEM with them in the aspect of bands distribution, accuracy, and consistency through experiments. Firstly, it is verified that the spatial dispersion of bands selected by clustering-based method, including SICEM, is better than that of ranking-based method. Then, via indexes of OA, AA, and KC, the results show that the comprehensive performance of SICEM is the best. Finally, we know that SICEM has good stability and can well adapt to the changes of external environment.

In practice, SICEM is a good solution if higher accuracy and less training costs are required simultaneously. The proposed algorithm provides an effective way to reduce the dimensions of samples, and meanwhile it keeps vital information for machine recognition. Besides BS, SICEM also fits some applications where the samples have two or more types of features, so that the hierarchical selection can be conducted through different perspective. Hence, it is a meaningful work to migrate the idea of algorithm to some traditional and emerging fields.

Although lots of works have been done to improve the capability of BS, there are still many technical obstacles that need to be overcome in the future. Henceforth, we will mainly take the following aspects as directions of innovation, including computation complexity decreased, accuracy, stability, and robustness improved, and adaptability enhanced to large-scale and high-dimensional data.

## Figures and Tables

**Algorithm 1:**
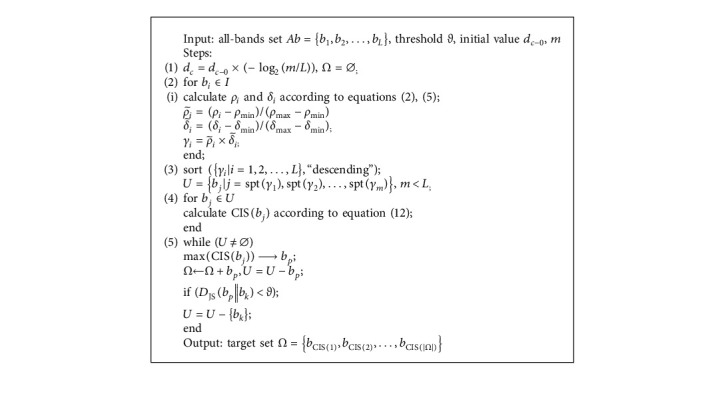
Implementation steps of SICEM.

**Table 1:** Essential situations of the experimental dataset.

Data set	Resolution	Pixels (background + object)	Spectral coverage range (*μ*m)	Bands	Available bands	Class
IndianP	145 × 145	21025 (10776 + 10249)	0.4∼2.5	220	200	16
PaviaU	610 × 340	2207400 (2164624 + 42776)	0.43∼0.86	115	103	9

**Table 2:** Classification accuracies (%) achieved using 40 selected bands on IndianP with KNN classifier.

Class	Algorithm				
	MVPCA	WaLuDi	DBSCAN	I-FDPC	SICEM
Corn-notill	66.5	71.3	69.6	71	73.7
Corn-mintill	55.2	64.4	69.4	70.8	70.8
Grass-pasture	82.6	88.7	86.2	85	84.4
Grass-trees	93.1	94.5	95	94.3	96
Hay-windrowed	97.6	96.3	96.6	95.8	96.6
Soybean-notill	62.5	76.2	73.7	68.3	75.7
Soybean-mintill	77.4	79.3	81.5	82.6	84.2
Soybean-clean	57.1	71.6	74	76.2	76
Woods	93.6	94.4	93.3	93.5	95.8
Buildings-Grass-Trees-Drives	54.7	61.7	63.9	57.4	63.6
AA	74.1	79.8	80.3	79.5	81.7
OA	80.2	83.1	82.5	83.3	86
Variance	287.9	166.5	141.4	165.9	135.3

**Table 3:** Classification accuracies (%) achieved using 21 selected bands on PaviaU with SVM classifier.

Class	Algorithm				
	MVPCA	WaLuDi	DBSCAN	I-FDPC	SICEM
Asphalt	86.5	87.6	87.7	89.8	91.7
Meadows	96.4	96.8	96.2	94.8	96.2
Gravel	72.7	71.3	75.8	74.2	77.1
Trees	84.2	91.3	91	92.1	93.5
Painted metal sheets	99.1	99.4	99.4	97.8	99.3
Bare soil	53.2	82.5	81.5	82.4	83.8
Bitumen	79.8	83.8	81.7	76.6	83.4
Self-Blocking Bricks	83.4	85.2	86.3	83.8	85.1
Shadows	97.2	98.5	98.5	98	98.7
AA	83.6	88.4	88.6	87.7	89.9
OA	85.4	89.3	89.1	89.3	90.5
Variance	206.9	82.6	68.3	78.9	61.0

**Table 4:** Iteration rounds correspond to different threshold intervals.

*ϑ*	Dataset/|*U*|	
	IndianP/80 bands	PaviaU/40 bands
[0, 0.04)	80∼76	40∼37
[0.04, 0.08)	77∼72	38∼34
[0.08, 0.12)	73∼67	37∼32
[0.12, 0.16)	71∼67	35∼31
[0.16, 0.2)	69∼62	35∼31

## Data Availability

Data used to support the findings of this study are included within the paper.
